# The role of neutrophils in tPA thrombolysis after stroke: a malicious troublemaker

**DOI:** 10.3389/fimmu.2024.1477669

**Published:** 2024-11-13

**Authors:** Qingcan Li, Jiao Ye, Zhifang Li, Qinghui Xiao, Senwei Tan, Bo Hu, Huijuan Jin

**Affiliations:** Department of Neurology, Union Hospital, Tongji Medical College, Huazhong University of Science and Technology, Wuhan, China

**Keywords:** neutrophils, thrombus, immunity, therapy, thrombolysis

## Abstract

Acute ischemic stroke represents a critical, life-threatening condition affecting the central nervous system. Intravenous thrombolysis with tissue plasminogen activator (tPA) remains a cornerstone for achieving vascular recanalization in such patients; however, its therapeutic utility is limited, with only approximately 10% of patients benefiting due to the narrow therapeutic window and significant risk of hemorrhagic transformation. Enhancing the efficacy of tPA thrombolysis is therefore imperative. Neutrophils have been identified as key modulators of thrombolytic outcomes, interacting with tPA post-stroke to influence treatment effectiveness. The binding of tPA to low-density lipoprotein receptor-related protein 1 (LRP-1) on neutrophil surfaces induces degranulation and formation of neutrophil extracellular traps (NETs). Conversely, neutrophils impede the thrombolytic action of tPA by obstructing its interaction with fibrin and activating platelets. These findings suggest that targeting neutrophils may hold promise for improving thrombolysis outcomes. This review explores the role of neutrophils in tPA-mediated thrombolysis following acute ischemic stroke, examines neutrophil-associated biomarkers, and outlines potential strategies for enhancing tPA efficacy.

## 1 Introduction

Acute ischemic stroke is a central nervous system (CNS) disorder characterized by high rates of morbidity, disability, mortality, and recurrence (1). According to the World Stroke Organization, stroke remains the second leading cause of death worldwide (2). Currently, the only intravenous thrombolytic therapies for acute ischemic stroke approved by the United States Food and Drug Administration are tissue plasminogen activator (tPA) agents, including recombinant tPA (Alteplase) and TNK-tPA (Tenecteplase). Nevertheless, effective treatment is achieved in only approximately 10% of patients due to the narrow therapeutic window and substantial risk of hemorrhagic transformation (HT) (1). Moreover, over half of treated patients derive no significant benefit, with recanalization rates remaining low at 20%-23.6% (3, 4). Therefore, improving the efficacy of tPA-mediated thrombolysis is a pressing clinical need.

Although the brain was once considered to possess immune privilege, accumulating evidence indicates that peripheral immune cells actively participate in intracranial inflammation following stroke (5). Among these immune cells, neutrophils are the first-line responders recruited to the affected brain tissue (6). Their numbers markedly increase in the brain after stroke onset, and significant neutrophil infiltration is also observed within thrombi (7, 8). Neutrophils contribute to neurovascular unit damage by releasing various deleterious molecules (8, 9). Additionally, tPA can directly modulate neutrophil activity, exacerbating brain injury (10). Neutrophils also impair the thrombolytic efficacy of tPA by obstructing its interaction with fibrin and promoting platelet activation, while neutrophil extracellular traps (NETs) within the thrombus further contribute to tPA resistance (11–13). Consequently, a deeper understanding of the interactions between neutrophils and tPA is essential for enhancing the safety and effectiveness of thrombolytic therapy.

This review focuses on the interplay between neutrophils and tPA during thrombolysis. It first examines the impact of neutrophils on the outcomes of tPA therapy for acute ischemic stroke, then summarizes neutrophil-associated biomarkers that may predict thrombolytic prognosis, and finally proposes potential therapeutic targets for improving the efficacy of tPA thrombolysis.

## 2 The mobilization of neutrophils after ischemic stroke

Following the onset of stroke, neutrophil levels rapidly rise across various compartments, including peripheral circulation, bone marrow, spleen, and even the brain. Neutrophil counts in the peripheral blood increase for the first three days post-stroke, subsequently returning to baseline within 3-7 days (14). This surge is primarily attributed to the mobilization of bone marrow and splenic stores, driven by the activation of the sympathetic nervous system and the hypothalamic-pituitary-adrenal axis (15, 16). In the bone marrow, neutrophil levels elevate as early as 10 minutes to 4 hours post-stroke, decline by 12 hours, and then continue to rise, persisting for at least seven days (14). Similarly, splenic neutrophils increase between 6-12 hours, peak at 12-24 hours, and revert to baseline within 2-7 days (17). Notably, the skull also serves as a hematopoietic source of neutrophils, with cerebrospinal fluid conveying injury signals to the skull bone marrow to stimulate neutrophilogenesis and mobilization (5). The pro-inflammatory cytokines and chemokines secreted by resident brain cells facilitate the infiltration of peripheral neutrophils into the brain parenchyma (14). Within two hours of stroke onset, neutrophils begin rolling and adhering to cerebral pial vessels, proceeding to infiltrate the brain tissue between 6-8 hours (18). Consequently, brain neutrophil numbers surge within the first day, peaking at 1-3 days, followed by a gradual decline from days 4 to 7 (14). Administration of tPA has been shown to further elevate neutrophil counts (19), with a 31% increase in circulating neutrophils observed as early as one hour post-infusion (20).

Upon reaching cerebral vessels, neutrophil adhesion is initiated by interactions between P-selectin glycoprotein ligand-1 (PSGL-1) on neutrophils and endothelial P- and E-selectin, anchoring neutrophils to the endothelium. Subsequent engagement of lymphocyte function-associated antigen 1 (LFA-1) and PSGL-1 with endothelial E-selectin and intercellular adhesion molecule-1 (ICAM-1) facilitates the slow rolling of neutrophils along the endothelium. Arrest and firm adhesion are mediated by integrins macrophage-1 antigen (MAC-1)/LFA-1 and endothelial ICAM-1/2 interactions. Neutrophils then crawl along the endothelium *via* MAC-1 and ICAM-1/2 before transmigrating into the brain parenchyma (18). Evidence suggests that tPA enhances neutrophil migration to brain vasculature *via* the annexin 2-MAPK pathway (20). Additionally, tPA may function as a cytokine by binding to the LRP-1 receptor, activating the cAMP/PKA signaling cascade, and inducing ICAM-1 expression in cerebral microvascular endothelial cells, potentially increasing neutrophil adhesion and transmigration (21).

## 3 Effect of neutrophils on tPA thrombolysis treatment

**Figure 1:**
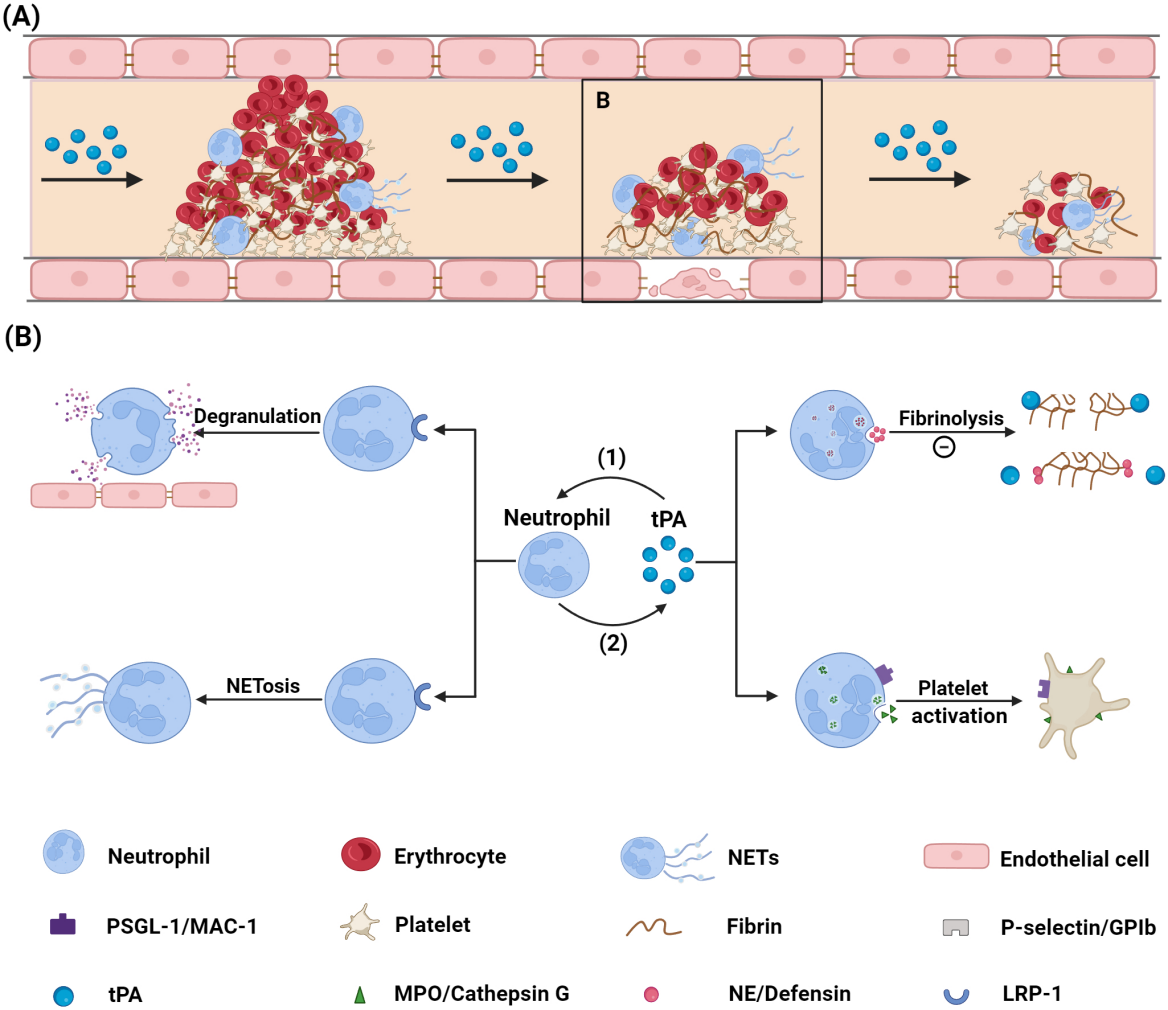
Mechanism *via* which neutrophils influence tPA thrombolysis. **(A)** Diagram illustrating the thrombolysis process. **(B)** Diagram showing the interaction between neutrophils and tPA: (1) tPA binds to the LRP-1 receptor on neutrophil surfaces, inducing neutrophil degranulation and NETs formation; (2) Neutrophils impair tPA’s thrombolytic efficacy by preventing tPA-fibrin binding through the release of NE and defensin, and by activating platelets *via* ligand-receptor interactions. tPA, tissue plasminogen activator; PSGL-1, P-selectin glycoprotein ligand-1; MAC-1, macrophage-1; NE, neutrophil elastase; NETs, neutrophil extracellular traps; GPIb, glycoprotein Ib; MPO, myeloperoxidase; LRP-1, low density lipoprotein receptor-related protein 1. Created with BioRender (BioRender.com/r33o076).

Neutrophils contribute not only to thrombus formation during stroke but also influence vascular recanalization following thrombolytic therapy, while potentially exacerbating complications after thrombolysis (22, 23). The following sections explore the distinct roles of neutrophils and NETs in thrombolysis as shown in **Figure 1**.

### 3.1 Neutrophils impaired the vascular recanalization after thrombolysis

Leukocytes, particularly neutrophils, are ubiquitous in ischemic stroke thrombi (24). They constitute approximately 4% of the cellular content within thrombi and occupy 0.5-20% of the thrombus area, with neutrophils being the predominant leukocyte type (22, 24). Di Meglio et al. performed *ex vivo* thrombolysis on the thrombi retrieved *via* thrombectomy, revealing that platelets, fibrin, and leukocytes were the main components of unresolved thrombi, suggesting their adverse impact on thrombolytic efficacy (25). Furthermore, a retrospective study of 92 patients with anterior circulation stroke who underwent combined intravenous thrombolysis and endovascular thrombectomy demonstrated that a higher neutrophil content in thrombi was significantly correlated with increased resistance to tPA (11).

Immunohistochemical analyses have shown that stroke thrombi are composed predominantly of platelet-rich regions interspersed with red blood cell-rich areas (7). Neutrophils tend to localize within platelet-rich regions or at the interfaces of these zones, likely due to their close interactions with platelets (7). Notably, thrombi feature a platelet-rich outer shell, which is particularly resistant to tPA-mediated thrombolysis (25, 26). Neutrophils may enhance thrombolytic resistance by promoting platelet activation and aggregation through ligand-receptor interactions or secretion of soluble mediators. PSGL-1, a mucin-like protein on neutrophils, binds to the platelet P-selectin receptor, leading to platelet integrin activation and promoting platelet aggregation (27, 28). Additionally, neutrophil MAC-1 can interact with platelet glycoprotein Ib (GPIb), forming neutrophil-platelet aggregates and inducing P-selectin expression and integrin activation *via* GPIb-mediated Akt phosphorylation (29). The stabilization of platelet-neutrophil interactions is further supported by other ligand-receptor signals, including GPVI, extracellular matrix metalloproteinase (MMP) inducers, ICAM-2, and LFA-1 (30).

Neutrophils also release soluble factors that activate platelets, thereby enhancing platelet aggregation and activating the coagulation cascade (31). Myeloperoxidase (MPO), predominantly secreted by neutrophils, has been shown to induce partial platelet activation and may increase platelet mechanical strength by remodeling the actin cytoskeleton, which further promotes aggregation (32, 33). The neutrophil granule enzyme cathepsin G has been implicated in platelet activation in experimental models of mesenteric arteriolar thrombosis and transient middle cerebral artery occlusion (tMCAO) (34).

Upon interacting with platelets, neutrophils release extracellular vesicles containing arachidonic acid, which can be taken up by platelets and utilized as a substrate for cyclooxygenase 1 (COX-1) (35). COX-1 metabolizes arachidonic acid to produce thromboxane A2, a potent paracrine factor that amplifies platelet aggregation and activates endothelial cells (36). Conversely, platelets can secrete interleukin-1β (IL-1β), an inflammatory cytokine that induces a proinflammatory state in neutrophils (37). Platelets also release neutrophil-activating peptide-2, facilitating neutrophil recruitment to the thrombus (38). This bidirectional activation establishes a self-perpetuating cycle of neutrophil and platelet activation.

Following ischemic stroke, neutrophils secrete various granule proteins, such as neutrophil elastase (NE) and defensin, which have been shown to impair tPA-mediated fibrinolysis. NE, a neutral serine protease found in neutrophil azurophil granules, interferes with tPA activity by degrading proteins necessary for its function, including fibrin and extracellular matrix components (39). Specifically, NE degrades the fibrin α-chain, a crucial component in tPA-mediated fibrinolysis. Incubation with NE diminishes fibrin’s ability to facilitate plasminogen activation by tPA, thereby significantly impairing the fibrinolytic process (40). Moreover, *in vitro* studies indicate that NE can degrade plasminogen directly, inhibiting its conversion to plasmin and leading to fibrinolytic resistance (41). This phenomenon also occurs *in vivo*, limiting plasmin production and hampering thrombus dissolution post-tPA administration (41). Defensin, another protein present in neutrophil homogenates with a molecular weight below 13 kDa, has antifibrinolytic properties due to its competitive binding with plasminogen for fibrin and tPA binding sites (42, 43). However, the specific impact of NE and defensin on tPA thrombolysis after ischemic stroke remains unclear.

Even after successful large-vessel recanalization with thrombolysis, full restoration of blood flow may be compromised by the no-reflow phenomenon, a condition attributed to neutrophils (44, 45). Neutrophils, when activated, exhibit reduced deformability, impeding their passage through narrow capillaries and causing microvascular obstruction (46). Additionally, extensive adhesion of neutrophils to endothelial cells in postcapillary venules increases microvascular resistance, further contributing to impaired reperfusion (44, 47).

### 3.2 NETs impaired the vascular recanalization after thrombolysis

NETs are highly negatively charged structures composed primarily of DNA, histones, and various proteins such as NE, MPO, and cathepsin G (48). The formation of NETs, termed NETosis, can occur through different pathways: suicidal NETosis, which relies on NADPH oxidase; vital NETosis, which is independent of NADPH oxidase; and mitochondrial NETosis (49). Under normal physiological conditions, the web-like architecture of NETs helps contain the spread of pathogens, with their adherent proteins exhibiting bactericidal properties (49). However, excessive NETs formation can aggravate inflammation and contribute to tissue injury (50).

The involvement of NETs in stroke pathology was first highlighted by Perez-de-Puig et al., who demonstrated that neutrophil activation following stroke initiates NETosis (51). Further studies by Vallés et al. showed significantly elevated plasma NETs levels in patients with acute ischemic stroke (52). Laridan et al. confirmed the presence of NETs within stroke thrombi, underscoring their significant role in the pathogenesis of ischemic stroke (53). NETs are predominantly found in the outer layers or platelet-rich cores of thrombi (7), and their presence has been linked to impaired tPA-mediated thrombolysis (54, 55). Experimental models have provided insights into the role of NETs in thrombolytic resistance. In a mouse photothrombotic stroke model, researchers induced thrombi consisting primarily of platelets and NETs. Administration of DNase I alone, which degrades the DNA backbone of NETs, led to successful recanalization of the occluded vessels (55). Additionally, DNase I enhanced tPA-induced thrombolysis of thrombi from patients with acute ischemic stroke ex vivo, although DNase I alone did not induce thrombolysis (54, 55). The disparity in results between these experiments likely reflects differences in thrombus composition. In the photothrombotic model, thrombi are densely packed with platelets and NETs, making them more susceptible to DNase I-mediated dissolution. In contrast, patient thrombi exhibit a more complex composition, with only certain regions rich in platelets and NETs, limiting the efficacy of DNase I as a standalone treatment for complete thrombus dissolution.

Mechanistically, the histones and DNA components of NETs contribute to thrombolytic resistance by altering clot structure and lysis dynamics: histones influence the architecture of the clot, while DNA modifies the degradation process (56). In thrombin-induced plasma clots, histones have been shown to disrupt the spatial organization of monomer blocks and protofibrils, resulting in an increased fibrin fiber diameter (57). Additionally, extracellular histones enhance thrombin generation, raising plasma thrombin levels and leading to the formation of finer fibrin fibers (58, 59). The overall effect of histones on fibrin structure is a combination of these opposing influences, which tends to increase fibrin fiber thickness (56, 60). Thicker fibrin fibers are generally associated with greater clot stability, potentially inhibiting fibrinolysis (61).

DNA within NETs further impairs tPA-induced clot lysis by altering the breakdown pattern, delaying complete clot dissolution (57). Normally, fibrin is cleaved into large fibrin degradation products (FDPs) with molecular weights greater than 150 kDa, facilitating clot resolution. However, DNA binds to FDPs, stabilizing the fibrin network and necessitating further cleavage of large FDPs into smaller fragments for complete lysis, thus delaying clot dissolution (56). Moreover, DNA reduces clot permeability due to its pore-filling properties, thereby inhibiting fibrinolysis (57). The antifibrinolytic effect of DNA may also arise from its interactions with fibrin and tPA (57, 62). DNA can compete with fibrin for plasmin binding, affecting the efficiency of fibrinolysis, and increase the susceptibility of tPA to inhibition by plasminogen activator inhibitor 1 (PAI-1), the primary endogenous inhibitor of tPA (62). Additionally, NE-DNA complexes present in NETs exhibit proteolytic activity, leading to the fragmentation of plasminogen, which decreases the local concentration of intact plasminogen while generating antifibrinolytic plasminogen fragments (63).

*In vitro* studies have demonstrated that NETs within thrombi contribute to thrombolytic resistance. However, the impact of circulating NETs on thrombolysis *in vivo* remains uncertain, as NETs may influence stroke outcomes through various mechanisms (49).

### 3.3 Neutrophils aggravated HT and edema after tPA thrombolysis

Thrombolysis-related complications include cerebral edema (CE), HT, and systemic hemorrhage in the acute phase (23). CE arises from plasma leakage across the compromised blood-brain barrier (BBB), leading to tissue swelling (64). The risk of severe, life-threatening edema is highest when the middle cerebral artery is occluded, often resulting in tissue displacement, elevated intracranial pressure, and potentially fatal outcomes within 2-5 days post-stroke (65). Recent observations by Frisullo et al. indicate that patients undergoing tPA treatment experience a more pronounced increase in CE during the first 72 hours after stroke onset, identifying tPA as a trigger for the onset and progression of CE (66). HT, another serious thrombolysis-related complication, can range from minor petechial bleeding to large parenchymal hemorrhages (PH) (67). Both types of HT arise from a combination of BBB disruption and the pharmacological effects of tPA (68). Given the role of neutrophils in BBB damage during cerebral ischemia and their interaction with tPA, neutrophils have been implicated in exacerbating these complications (68). Experimental evidence supports this association: in hypertensive rats, pretreatment with monoclonal anti-neutrophil antibodies reduced neutrophil infiltration and HT after thrombolysis, while induction of neutrophilia with granulocyte colony-stimulating factor increased the risk of bleeding in a mouse model of tMCAO (69, 70). Clinical data supporting these findings will be discussed subsequently.

Post-stroke, neutrophils exacerbate BBB disruption through the production of proteases such as MMPs and elastase, reactive oxygen species (ROS), lipocalin-2 (LCN-2), and various cytokines and chemokines (18, 71–73). tPA can prompt early neutrophil degranulation, releasing significant amounts of MMP-9 *via* the LRP-1/Akt or ERK1/2 signaling pathways in cultured neutrophils (10, 74). Moreover, tPA treatment enhances the enzymatic activity of MMP-9 released by neutrophils (75). The sudden influx of oxygen during reperfusion after tPA thrombolysis leads to excessive ROS production by neutrophils, causing oxidative damage to junction proteins (68, 71). This oxidative stress further compromises the neurovascular unit by damaging endothelial cells, smooth muscle cells, pericytes, and astrocytes, thereby increasing BBB permeability and the risk of HT (72). Additionally, tPA has been shown to stimulate neutrophils to overproduce NETs *via* the LRP-1/PAD4 pathway following ischemic stroke (8). NETs contribute to neuroinflammation by activating monocytes and macrophages, promoting the release of pro-inflammatory cytokines, and mediating inflammasome activation (49). Furthermore, NETs can directly compromise endothelial integrity by disrupting intercellular adhesion junctions and reorganizing the actin cytoskeleton, leading to microvascular leakage and impaired barrier function (76, 77).

## 4 Neutrophil-related biomarkers for thrombolysis prognosis

Given the critical role of neutrophils in thrombolysis, neutrophil-related biomarkers have significant potential as predictors of thrombolysis outcomes. Here, these biomarkers are categorized into three groups: dynamic changes in neutrophil numbers, neutrophil-associated parameters, and NETs-related components.

### 4.1 Dynamic changes in neutrophil numbers

Numerous studies have established the predictive value of circulating neutrophils for thrombolysis outcomes. Maestrini et al. reported that higher neutrophil counts at admission were independently associated with an increased risk of symptomatic intracerebral hemorrhage (sICH) and worse functional outcomes three months post-thrombolysis (78). However, other researchers argue that changes in neutrophil counts following thrombolysis provide a better prognostic indicator than baseline counts (19). For instance, a study showed that a 10% increase in neutrophil numbers after tPA administration was linked to an 83% higher risk of death or severe disability at three months (19). Ying et al. also found that dynamic increases in neutrophil counts post-thrombolysis were predictive of PH and poor outcomes at three months, whereas admission neutrophil levels were not reliable indicators (79). These discrepancies may stem from the confounding effect of infection in some patients upon admission, which influences neutrophil counts and affects the predictive accuracy for poor outcomes.

The neutrophil-to-lymphocyte ratio (NLR) has also emerged as a relevant biomarker for assessing thrombolysis prognosis. Elevated NLR at admission has been independently associated with sICH and early neurological deterioration post-thrombolysis (78, 80). Similar to neutrophil counts, some studies suggest that post-thrombolysis NLR is a stronger predictor of outcomes than admission NLR (81). For example, Guo et al. found no significant difference in admission NLR between patients with and without PH (82). Chen et al. showed that lower NLR levels, both at admission and after thrombolysis, were associated with favorable neurological outcomes, but NLR measured post-thrombolysis had superior discriminative ability for neurological prognosis (83). Additionally, a dynamic rise in NLR post-thrombolysis was predictive of PH, whereas baseline NLR was not (79).

Other neutrophil-related ratios, such as the neutrophil-to-high-density lipoprotein cholesterol ratio (NHR) and platelet-to-neutrophil ratio (PNR), have also been studied as prognostic markers. NHR levels were found to be higher in patients with acute ischemic stroke compared to healthy controls, with a positive correlation between NHR and the severity of neurological damage (84). Elevated NHR at 24 hours post-intravenous thrombolysis was significantly associated with poor outcomes (84). An inverse relationship was observed between PNR and stroke severity; lower PNR levels, whether at admission or 24 hours post-thrombolysis, were independently associated with unfavorable functional outcomes (85). Notably, PNR measured at 24 hours post-thrombolysis showed greater reliability in predicting poor prognosis compared to PNR at admission (85).

### 4.2 Neutrophil- associated parameters

Following a stroke, neutrophils are the primary source of MMP-9 in both peripheral blood and brain tissue (86, 87). MMP-9 has shown potential as a predictor of stroke prognosis, supported by clinical studies demonstrating that higher plasma MMP-9 levels are associated with more severe strokes and an increased risk of poor functional outcomes (88, 89). Additionally, elevated MMP-9 concentrations during the acute phase are independent predictors of HT across all stroke subtypes (90). Another study has also established a close relationship between plasma MMP-9 levels and the occurrence of PH following tPA treatment (91). A meta-analysis further supported MMP-9 as a sensitive and specific biomarker for predicting the risk of HT after thrombolysis or stroke (92). However, some studies present differing perspectives. Zheng et al. found that elevated plasma MMP-9 levels were associated with poor prognosis one year after stroke only in patients with dyslipidemia, not in those with normal lipid levels (93). Similarly, Costru-Tasnic et al. reported that while higher plasma MMP-9 levels at admission were linked to an increased risk of HT and worse neurological outcomes at discharge and three months, these associations did not reach statistical significance (94). This lack of correlation may be attributed to the timing of blood sample collection, which occurred around 21.9 hours post-thrombolysis—potentially too early to detect peak MMP-9 levels, which typically rise from 1-5 days after thrombolysis (95).

NE, a neutral serine protease contained in neutrophil azurophil granules, also plays a role in the breakdown of the BBB (18). Plasma NE levels have been found to be significantly elevated in patients with acute ischemic stroke compared to controls (96). High admission plasma NE levels have predictive value for identifying patients likely to experience poor functional outcomes three months after tPA thrombolysis (97).

### 4.3 NETs components

As previously discussed, NETs contribute to thrombolytic resistance and BBB disruption in stroke. Circulating NETs levels increased after ischemic stroke and were positively correlated with stroke severity (52, 98). Although most clinical research on NETs as a prognostic tool for thrombolysis remains limited, evidence from studies in related conditions, such as ST-segment elevation myocardial infarction, suggests that NETs could serve as independent predictors of outcomes, indicating their potential utility in stroke prognosis (99).

NETs components, including citrullinated histone 3 (citH3), nucleosomes, cell-free DNA (cfDNA), elastase, and MPO, are measurable in plasma and commonly used as NETs indicators. Histone citrullination mediated by peptidylarginine deiminase 4 (PAD4) is a crucial step in NETs formation (100). In patients with stroke, citH3 is present in almost all thrombi, co-localizing with extracellular DNA, thereby confirming the specific presence of NETs (53). CitH3 is regarded as a NETs-specific marker and has been independently associated with various conditions, including myocardial infarction, stroke, stent thrombosis, and cardiovascular death (49, 99). Vallés et al. found significant elevation of NETs markers, such as cfDNA, nucleosomes, and citH3, in the plasma of patients with stroke compared to healthy controls, with citH3 showing the highest increase (72%) (52). During a year-long follow-up, elevated citH3 levels were independently associated with all-cause mortality, suggesting its potential as a prognostic marker in acute ischemic stroke (52). Thålin et al. developed a reliable assay for quantifying nucleosomal citH3 in plasma, using a semi-synthetic calibrator and specific monoclonal antibodies to ensure high accuracy and low variability (101). However, citH3 only detects PAD4-dependent NETosis, limiting its utility for vital and mitochondrial NETosis, which are PAD4-independent (102).

MPO is another specific marker for neutrophils, with MPO-DNA complexes being characteristic of NETs. Elevated plasma levels of MPO-DNA and citH3 after stroke onset have shown positive correlations with stroke outcomes (103, 104). In a tMCAO mouse model, prophylactic treatment with a NETs-inhibitory peptide significantly reduced circulating MPO-DNA levels and cerebral infarct size (103). Measuring MPO-DNA *via* enzyme-linked immunosorbent assay (ELISA) is currently considered the most specific, objective, and quantitative method for assessing NETosis (102).

Circulating cfDNA, a degraded DNA fragment present in plasma, is another marker linked to neutrophil counts and injury severity in patients with acute ischemic stroke (105). However, since neutrophils or NETs are not the sole contributors to cfDNA, it serves only as a reference indicator of neutrophil or NETs activity rather than a specific marker (49). Vajpeyee et al. found that lower circulating cfDNA levels were significantly associated with improved outcomes after stroke (106), and admission levels have been reported as predictors of short-term neurological outcomes after intravenous thrombolysis (107). Combining MPO-DNA with cfDNA or citH3 assays enhances the assessment of NETs levels compared to individual markers alone (49). Despite these promising findings, the clinical application of NETs as biomarkers remains challenging, requiring further development, validation, and standardization to overcome current limitations (108).

## 5 Treatment targeting neutrophils

Neutrophils significantly influence tPA thrombolysis outcomes after stroke, and targeting these cells could potentially improve thrombolytic prognosis. However, directly targeting neutrophils poses challenges for clinical translation due to the risk of neutropenia-induced infections. Therefore, strategies focusing on the modulation of neutrophil recruitment and activation may offer a safer and more practical approach (68). Additionally, inhibiting NETs formation or promoting their degradation represents a promising therapeutic direction for stroke treatment (49).

### 5.1 Drugs targeting neutrophils

One such strategy involves UK-279,276, a recombinant glycoprotein with a selective affinity for the CD11b/CD18 integrin on neutrophils, which can inhibit neutrophil adhesion to endothelial cells and prevent their migration (109). In a rat model of tMCAO, combining tPA with UK-279,276 administered 4 hours post-ischemia significantly reduced ischemic damage without increasing the risk of HT (110). Although there is a theoretical concern that UK-279,276 could elevate infection risk, a single dose of up to 1.5 mg/kg was well tolerated in patients with acute stroke (109). Despite these promising preclinical results, the subsequent clinical trial was discontinued after UK-279,276 failed to demonstrate a significant improvement in outcomes for patients with acute ischemic stroke (111). Several factors may have contributed to this outcome. One potential reason is the difference between rats and humans regarding brain size, anatomy, and composition, which could limit the translation of results from animal models to clinical practice. Future experiments using primate models may provide insights that better support clinical applications. Additionally, the failure may reflect inherent limitations in the drug itself, as other agents targeting neutrophil adhesion and migration, such as R6.5 and Hu23F2G, were also discontinued following unsuccessful phase III clinical trials (112, 113).

Minocycline, a tetracycline antibiotic, is characterized by high lipid solubility, a broad antibacterial spectrum, an extended half-life, and efficient gastrointestinal absorption (114). Recent studies have highlighted its modulatory effects on neutrophils. Mechanistically, minocycline inhibits the respiratory burst and transendothelial migration of neutrophils, enhancing its anti-inflammatory properties in a dose-dependent manner (115). Additionally, it suppresses the production and activity of neutrophil-derived MMP-9 (116, 117). In rat models of tMCAO and focal embolic stroke, minocycline significantly reduced neutrophil infiltration and the tPA-induced increase in MMP-9 levels, without impairing fibrinolysis, leading to smaller infarct sizes and improved outcomes (118, 119). Furthermore, combining minocycline with tPA lowered the incidence of HT and extended the thrombolytic window to six hours post-stroke onset in tMCAO models (120). Clinical trials have also investigated its potential in acute ischemic stroke. In the Minocycline to Improve Neurological Outcome in Stroke (MINOS) trial, plasma MMP-9 levels decreased over hours to days in the minocycline-treated group, though the study did not specifically evaluate its impact on stroke outcomes (121). A meta-analysis suggested that minocycline may enhance stroke recovery, although no significant association was found with reduced HT risk (122). The combination of tPA and minocycline was well tolerated, with no notable adverse effects such as dizziness, gastrointestinal discomfort, or infusion reactions, confirming the safety of this therapeutic approach (123). These findings suggest that minocycline could be a promising adjunct to tPA in acute ischemic stroke treatment; however, larger, randomized clinical trials are needed to further validate its efficacy and safety.

Thiazolidinediones (TZDs), commonly used hypoglycemic agents, also exhibit anti-inflammatory and neuroprotective properties. Studies have shown that prophylactic or delayed administration of rosiglitazone following ischemia/reperfusion reduces neutrophil counts in both blood and brain parenchyma, alleviating neurological deficits and decreasing infarct volume in tMCAO models in mice and rats (124, 125). However, Cuartero et al. observed that rosiglitazone did not reduce overall neutrophil infiltration in the brain but instead accelerated their migration into the ischemic core (126). Rather than worsening ischemic injury, this accelerated infiltration provided neuroprotection and contributed to reduced infarct size (126). The underlying mechanism was attributed to an increase in N2 neutrophils, a subtype known for its protective effects in ischemic stroke (14, 126). Additionally, combining rosiglitazone with tPA has been shown to enhance neuroprotective outcomes and extend the therapeutic time window for tPA administration in focal embolic stroke models (127). Rosiglitazone also decreases the incidence of secondary HT associated with acute hyperglycemia in tMCAO rats, an effect independent of its glycemic control properties (128). A novel approach has been proposed for delivering TZDs to the brain using neutrophil-mediated uptake *via* bacteria-derived outer-membrane vesicles, demonstrating therapeutic efficacy in tMCAO mouse models (129). These experimental findings underscore the need for clinical trials to evaluate the potential translation of these therapies into clinical practice.

The protective effects of the discussed drugs primarily stem from inhibiting neutrophil recruitment and activation or promoting the transformation of neutrophils into the N2 subtype. However, these mechanisms may impair the anti-infective functions of neutrophils, raising concerns about increased infection risk. It is crucial for researchers to consider these side effects to prevent severe infections in patients undergoing treatment.

### 5.2 Treatment targeting NETs

Inhibiting NETs formation and promoting their degradation are promising strategies for stroke treatment (49). Although existing research suggests that targeting NETs can improve stroke prognosis, evidence directly linking NETs inhibition to better thrombolysis outcomes is lacking. Given the impact of NETs on thrombolysis, further investigation is warranted to determine whether targeting NETs could enhance thrombolytic efficacy.

#### 5.2.1 PAD4 inhibition

PAD4-mediated histone citrullination is a key step in NETosis. Inhibiting this process with chloramide, a PAD4 inhibitor, has been shown to restore blood flow to ischemic areas and reduce renal injury in ischemia/reperfusion models (49). GSK484, a highly specific and reversible PAD4 inhibitor, can block NETs formation and prevent neutrophil infiltration into tissues, thereby reducing neural damage following subarachnoid hemorrhage (130, 131). Similarly, GSK199 pretreatment decreased infarct size and improved neurological outcomes in tMCAO mouse models (103). Administration of neonatal NETs inhibitory factor, which targets PAD4, one hour post-stroke resulted in significant reductions in circulating and cerebral NETs, leading to decreased neuronal apoptosis and infarct volume (103, 132). Notably, PAD4-deficient mice maintain intact immune function, suggesting that PAD4 inhibitors are unlikely to increase susceptibility to bacterial infections, thus supporting their potential use in stroke therapy (133).

#### 5.2.2 ROS inhibition

Edaravone, a free radical scavenger with neuroprotective effects, has been shown to reduce ROS production in neutrophils during the acute phase of stroke (134). It also decreases NETs levels in the cortex of permanent MCAO mice, leading to improved neurological function (104). Clinical trials have demonstrated that edaravone reduces serum NETs levels in patients with stroke, enhances early recanalization, and improves 90-day functional outcomes (104, 135, 136).

Vitamin C, an ROS inhibitor, has been found to reduce NETs formation in activated neutrophils *in vitro* (137). In a tMCAO model, it crosses the BBB as dehydroascorbic acid and exerts neuroprotective effects (138). However, clinical studies on vitamin C’s role in stroke prevention yield inconsistent results; some suggest that long-term dietary vitamin C intake lowers the risk of ischemic stroke, while others find no significant effect (139). These discrepancies may be due to its benefits being limited to specific populations, underscoring the need for future well-designed clinical trials.

#### 5.2.3 NETs degradation

Promoting NETs degradation offers a promising approach for stroke therapy. Experimental evidence indicates that DNase I reduces BBB disruption and enhances post-stroke neovascularization in permanent MCAO mouse models (104, 140). As previously discussed, combining DNase I with tPA facilitates thrombus dissolution, potentially improving recanalization outcomes (53–55). However, DNase I specifically degrades extracellular DNA and does not target histones, which remain bound to the blood vessel walls post-treatment and may exert cytotoxic effects (141, 142). To address this limitation, activated protein C (APC) has emerged as a complementary therapeutic strategy. APC can enzymatically cleave histones, thereby neutralizing their toxicity. In dose-dependent studies, APC has been shown to reduce infarct volume and confer neuroprotection in tMCAO models (143, 144). Moreover, a clinical study demonstrated that 3K3A-APC, a modified form of APC, exerts vascular protective effects, significantly reducing the risk of HT (145).

## 6 Conclusion

In conclusion, the interaction between neutrophils and tPA significantly influences the effectiveness of intravenous thrombolysis following a stroke. Upon administration, tPA mobilizes neutrophils to the brain parenchyma, amplifying their pathological impact. Neutrophils alter thrombus composition and affect the thrombolytic process, while also exacerbating BBB disruption, thereby increasing the risks of HT and cerebral edema. Additionally, elevated levels of neutrophil-related biomarkers are strongly associated with poorer outcomes after tPA thrombolysis. Several neutrophil-targeting drugs have shown potential in improving thrombolysis outcomes in animal models, though further clinical trials are needed to validate these findings. Thus, targeting neutrophils represents a promising strategy to enhance the therapeutic efficacy of tPA thrombolysis. Continued translational research is crucial to bridge the gap between experimental results and practical clinical applications.
